# Subdomains of executive function correlate with accuracy on a change detection task

**DOI:** 10.3758/s13423-026-02937-0

**Published:** 2026-06-08

**Authors:** Ashley Norman, Russell Conduit, Robin Laycock, Stephen R. Robinson

**Affiliations:** School of Health and Biomedical Sciences, RMIT University Bundoora Campus, Plenty Road, Bundoora, Vic 3083 Australia

**Keywords:** Change blindness, Change detection, Executive function, Cognitive ability, Processing speed

## Abstract

Change blindness involves a failure to detect changes in a visual scene, despite the differences being available to visual perception. Although change detection is crucial for many every day or professional behaviours (e.g., driving, occupations such as radiology), little is known about the individual differences that contribute to change detection ability or the cognitive domains involved. Previous research has implicated attentional and visual memory processes in successful change detection. The present study used a naturalistic change detection task, the Alternate Forms Flicker Task (AFFT), paired with a battery of nine cognitive tests, to ascertain which cognitive domains are most strongly associated with change detection accuracy in a sample of 260 participants. Strongest correlates with AFFT accuracy were tasks assessing top-down attentional search (Visual Attentional Capture and Control Task), visuospatial ability and memory (Austin Maze and Object 2back Task), and visual inspection time (Subtle Cognitive Impairment Test). Principal Axis Factoring extraction with Promax rotation showed that cognitive flexibility, visuospatial working memory, and attention and processing speed collectively accounted for 41% of the variability in test battery scores. Using these three factors, a multiple linear regression model significantly accounted for 10.0% of the variability in AFFT scores. Visuospatial working memory was the principal factor, indicating that individual differences in visuospatial working memory likely contribute to differences in change detection accuracy.

**Supplementary Information:**

The online version contains supplementary material available at 10.3758/s13423-026-02937-0.

## Introduction

**Fig. 1:**
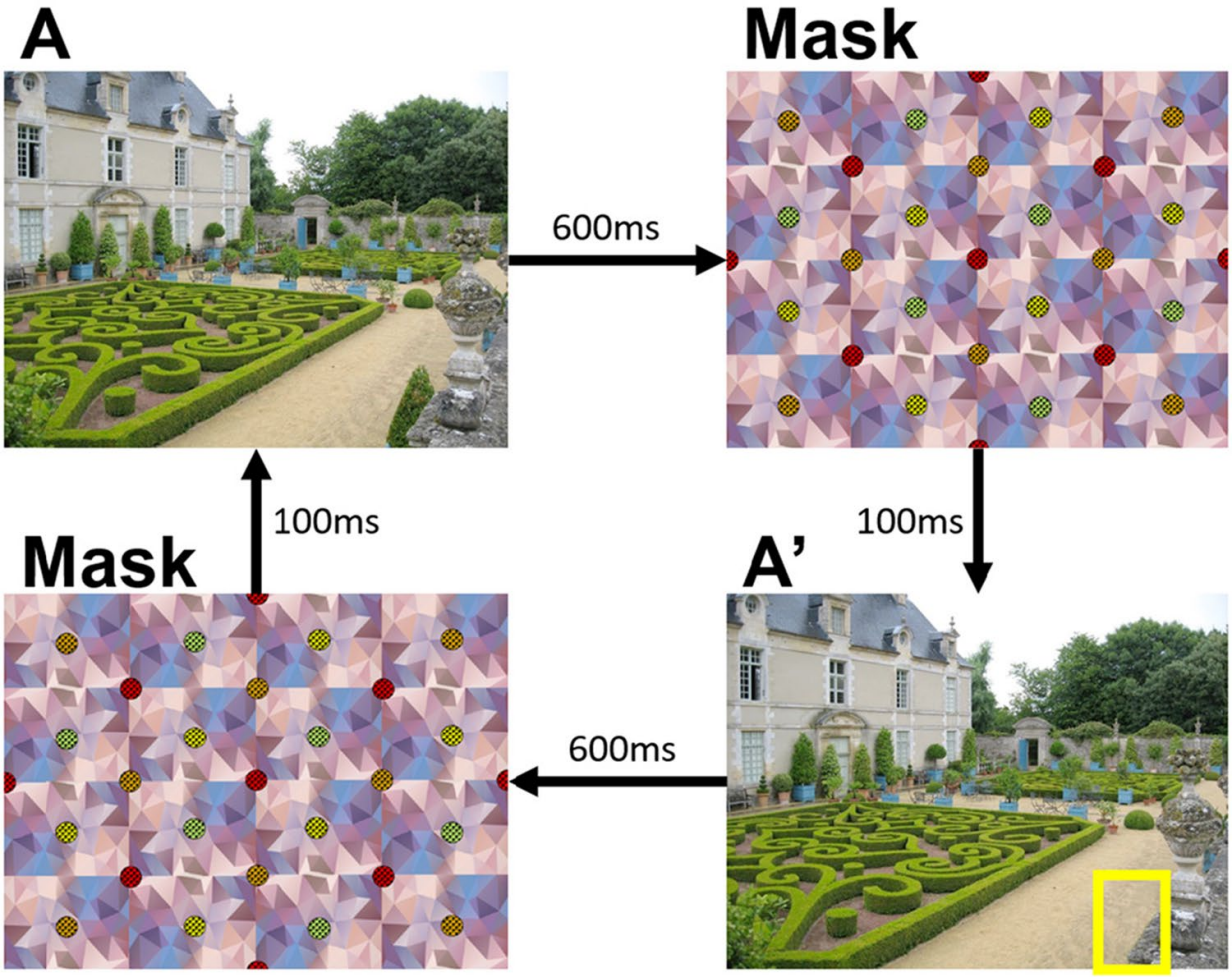
An example of a change blindness flicker paradigm. Whilst display durations vary between Change Blindness flicker paradigms, all involve the task alternating from an image to a mask, to an altered image, to a mask, and then back to the original image, where the cycle continues. In this example, the target to identify is the plant that appears and disappears in each cycle. The yellow box on image A is for illustrative purposes only

Change blindness (CB) refers to a phenomenon where individuals fail to detect visual changes despite the differences being available to visual perception and obvious when pointed out (Rensink et al., 1997). Whilst different methods exist to study this phenomenon (e.g., one-shot, mud-splash), one method of investigating CB is the ‘flicker paradigm’ (Fig. 1). This method simulates an eye saccade or blink by showing two images in succession interrupted by a brief flicker of a blank/grey mask that acts as global transient, blocking the perception of movement that would otherwise be generated by the changing feature (Jensen et al., 2011; Rensink et al., 1997).

Successful change detection (CD) requires a range of cognitive skills, with previous studies highlighting the important contribution of executive functioning, including attentional processes (Jensen et al., 2011; Pringle et al., 2001; Rensink et al., 1997; Rizzo et al., 2009; Simons & Rensink, 2005), and working memory (Colflesh & Wiley, 2013; Huang et al., 2012; Jensen et al., 2011; Kerrigan et al., 2017; Tseng et al., 2010). Without focussed attention to the target, there is no conscious awareness of the change, and thus individual differences in attention control should predict CD (Cohen et al., 2012; Jensen et al., 2011). However, inattention cannot be the sole reason for CB, as it can occur even if attention is directed on the object that changes both pre- and post-change (Caplovitz et al., 2008; Jensen et al., 2011; Simons & Levin, 1998). Instead, successful CD also requires working memory processes to encode and compare visual representations of the changing feature over time (Jensen et al., 2011; Rizzo et al., 2009; Simons & Ambinder, 2005). Thus, CB could occur due to either the observer never attending to the target, or a failure of visuospatial working memory at the encoding or comparison stage (File et al., 2022; Jensen et al., 2011). However, there is currently no consensus on which of these processes fails in CB (File et al., 2022).

Both attention control and working memory are important constructs for measuring individual differences in cognitive performance (Burgoyne et al., 2023; Draheim et al., 2021; Robison & Unsworth, 2017). Whilst they are closely related and support one another – sharing approximately 50% of their variability in individual differences – they are distinct cognitive constructs (Garner & Robison, 2025). However, individual differences in both constructs may be partially explained by processing speed (Cepeda et al., 2013), as it is strongly correlated with attention control (Burgoyne et al., 2023), and may account for individual differences in performance on executive function tasks (Löffler et al., 2024). Given that the CB flicker paradigm involves transiently presented stimuli interrupted by a brief flicker of a mask, processing speed should predict CD performance (Jensen et al., 2011; Wright et al., 2018). Consistent with this, slower processing speed has been associated with increased CB (Rizzo et al., 2009), and slower detection in a driving-related CD task (Batchelder et al., 2003; Pringle et al., 2001). Thus, examining how processing speed interacts with attention control and visuospatial working memory in predicting CD performance may provide further insight into the cognitive mechanisms involved.

Another important measure of individual differences is cognitive flexibility, which reflects the ability to mentally switch between cognitive strategies in response to changing demands of the environment (Diamond, 2013; Figueroa & Youmans, 2012; Hohl & Dolcos, 2024; Scott, 1962). Cognitive flexibility is required to prevent perseveration on a feature or attribute that is no longer relevant to the demands of a task (Introzziab et al., 2015). Cognitive flexibility is related to fluid intelligence (Colzato et al., 2006), which is thought to stem from working memory capacity. This relationship is said to be causal, with individuals having a larger working memory capacity being better able to manipulate and generate novel information (Draheim et al., 2022). However, the relationship between cognitive flexibility and CD performance is mixed, with some studies reporting that better cognitive flexibility is related to better CD performance (Rizzo et al., 2009; Youmans et al., 2011), and others finding no significant relationship (Huang et al., 2012). The inconsistent relationship between performance on tasks of cognitive flexibility and CD make it difficult to understand the role that cognitive flexibility plays in CD performance, highlighting the need for further investigation.

Despite CB being extensively researched over the past few decades, little is known about the individual differences that contribute to one’s ability to detect changes (Andermane et al., 2019). One possible explanation is that many studies investigating CB have used CD tasks that are suboptimal for the detection of individual differences. Many studies investigating CB had accuracy scores in excess of 80%, with standard deviations around 10% or often not reported (Andermane et al., 2019; Ashwin et al., 2017; Boot et al., 2009; Edwards et al., 2022; Fletcher-Watson et al., 2012; Kerrigan et al., 2017; Pringle et al., 2001; Sareen et al., 2016). Whilst this minimises between-participant variance (Draheim et al., 2019; Hedge et al., 2018), the limited variability curtails its usefulness when attempting to correlate CD scores with other cognitive measures (Draheim et al., 2022; Eledum, 2017; Rouder & Haaf, 2019). The restricted range of accuracy scores may have led researchers to focus on time taken to identify the target as the primary dependent variable. However, response time (RT) is a noisier variable than accuracy (Brysbaert, 2024), and participants who are prone to respond quicker and make more errors may appear to be better on the task than those who prioritised accuracy over speed (Draheim et al., 2021). In general, RT outcomes show greater trial-to-trial variability and are more prone to speed-accuracy trade-offs and other strategic influences not related to the construct of interest (see Draheim et al., 2019, for a review). Consequently, some researchers have opted to utilise accuracy-based outcomes for cognitive tasks rather than RT, as they tend to be more reliable and improve intercorrelations between other tasks and latent factor scores (Draheim et al., 2019, 2021; Snijder et al., 2024). Whilst studies investigating CB have reported detection rates around 50% with a reasonably wide spread of accuracy scores, of these studies, one involved an older population that included participants with early Alzheimer’s disease (Rizzo et al., 2009), while the other focussed on visual attentional processes (Huang et al., 2012). The present study used a CD flicker task that avoids ceiling and floor effects and produces a wide spread of accuracy scores. The discriminatory power of this task has enabled us to compare individual differences in CD accuracy with individual performance on a battery of tests that encompass the cognitive domains associated with executive functioning and processing speed.

In summary, the current literature indicates that attention control, working memory, cognitive flexibility, and processing speed are involved to some extent in CD, but their precise interplay remains unclear. The aim of the present paper was to investigate the contributions made by these cognitive processes to accuracy on a CD flicker task that is suitable for measuring individual differences. It was hypothesised that performance on a battery of nine tests that measure subdomains of executive function and processing speed would be significantly correlated with CD accuracy. This hypothesis was tested with an exploratory factor analysis followed by a multiple linear regression analysis.

## Material and methods

We report how we determined our sample size, all data exclusions (if any), all manipulations, and all measures in the study (Simmons et al., 2012). Data and materials for all experiments are freely available online (see Online Supplementary Materials).

### Participants

The 260 participants (107 males, 150 females, three others), aged 18–57 years (*M* = 25.3, *SD* = 7.5) in this study were recruited from a pool of participants who had previously completed a battery of tests in a larger experiment. Although no a priori sample size calculation specific to an exploratory factor analysis was conducted, a minimum sample size of 170 meets published guidelines to conduct an exploratory factor analysis on the theoretical expectation that a small number of factors would emerge with moderate-to-high communalities (Mundfrom et al., 2005). Whilst the exact number of factors and item loadings cannot be known a priori, it was anticipated that three factors would emerge representing each of the core executive functions with three to five items (based on the outcome variable(s) for each task). However, the final number of factors retained was determined empirically based on standard procedures (see Results). Inclusion was limited to participants who were aged 18–60 years; were not diagnosed with bipolar disorder, schizophrenia, attention-deficit hyperactivity disorder, or autism spectrum disorder; had no physical disability preventing them from responding quickly and accurately (e.g., paralysis due to a stroke); and had no sleep disorders based on self-report.

### Materials

#### Alternate forms flicker task (AFFT)

A flicker paradigm of the task influenced by preceding CB paradigms (Rensink et al., 1997; Sareen et al., 2016) was used for this study. Participants were shown two images that were identical except for the absence of a target object that was removed using Adobe Photoshop. This target is the change that participants needed to identify in each trial. These images were shown for 600 ms and alternated between one another with a visual mask interposed between the images for 100 ms (Fig. 1). The image bank for this task was purpose-built for the study to be more suitable for measuring individual differences (see Online Supplementary Materials). Images were presented in random order, and the initial presentation (Image A in Fig. 1) was randomised to be either the target-absent or target-present version of the image. Participants searched for the change and indicated it by clicking on its location with the mouse. Participants could click on the target location regardless of whether the current alternation was showing the target-present or target-absent version of the image.

Participants were randomly allocated to one of two versions of the task that each had a different library of images. These were known as Set A and Set B, with both sets having 19 images matched for difficulty. The complete image set was split into thirds, based on empirical image difficulty data derived from a preceding pilot study that involved a 40-image version of the set ***–*** providing a total of 80 images for analysis. Images with a detection rate 30% and under were classified as hard, images with a detection rate between 30.1% and 69.9% were classified as medium, and images with a detection rate of 70%+ were classified as easy (see Online Supplementary Materials). Participants had 30 s to identify the target and were instructed to prioritise accuracy over speed. If the participant did not find the target within the 30 s, this trial was scored as incorrect. Four additional images were used and purposely made to have easily identifiable targets and were shown after the fifth, 11th, 18th, and 22nd presentation. These images were shown to all participants and included as part of the participant feedback score shown at the end of the task; however, their performance on these four images was not used for the purpose of analysis. The dependent variable was the number of correct detections from the image set, when given a viewing limit of 30 s per image.

#### Tests of attentional subdomains

Three tasks were used to assess attention, attention control, and speed of response.

##### Flanker arrow task

**Fig. 2:**
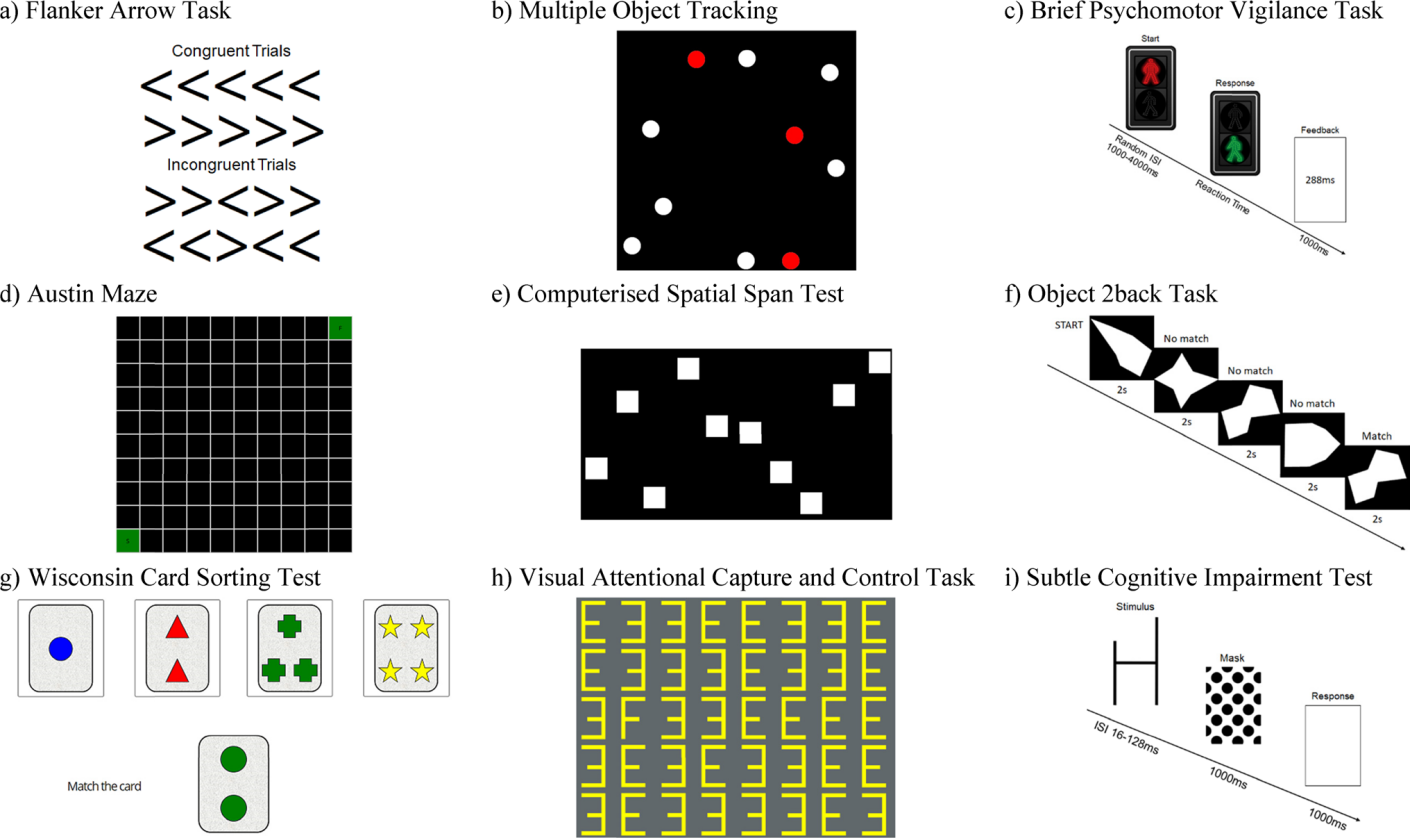
Executive function tasks used in the battery of testing. **a)** Flanker Arrow Task. **b)** Multiple Object Tracking. c**)** Brief Psychomotor Vigilance Task. **d**) Austin Maze.** e**) Computerised Spatial Span Test. **f**) Object 2back Task. **g**) Wisconsin Card Sorting Test. **h**) Visual Attentional Capture and Control Task. **i**) Subtle Cognitive Impairment Test

This task, widely considered to be a relatively ‘pure’ measure of executive function, involving selective attention, inhibitory control, conflict resolution, and processing speed, was adapted from Draheim et al. (2021). Participants were shown five arrows in the centre of the screen pointing either left (< < < < <) or right (> > > > >). Participants were instructed to push either the ‘left’ or ‘right’ arrow key on their keyboard corresponding to the direction of the middle arrow. Trials were either congruent where all arrows pointed in the same direction, or incongruent where the middle arrow pointed in the opposite direction to the flanking arrows (i.e., < < > < <; Fig. 2a). The task began with five practice trials with on-screen instructions, feedback of response, and an unlimited time to respond. The next block of ten practice trials provided no on-screen instructions or feedback, and participants had 800 ms to respond. In the testing stage, ten blocks of 18 trials (total 180 trials) were administered where the duration of the RT window to the stimulus presentation adapted to the participant’s performance. Beginning at 800 ms, the response deadline either decreased or increased depending on whether the participant was correct on 15 or more trials for each block. Each block contained 12 congruent and six incongruent trials with a randomised inter-stimulus interval of between 400 ms and 700 ms. Trials with no response (i.e., timed out) were counted as incorrect. The deadline to respond either increased or decreased by 90 ms in blocks 1**–**3, 30 ms in blocks 4**–**7, and 10 ms in blocks 8**–**10. The dependent variable was the final deadline value after the tenth block (i.e., what the duration to respond would have been on a hypothetical 11th block) as suggested by Draheim et al. (2021).

##### Multiple object tracking (MOT) task

This task involves visual attention, working memory, inhibitory control, and processing speed, and was modified from existing MOT paradigms (Meyerhoff & Papenmeier, 2020; Souza & Oberauer, 2017; Zwierko et al., 2022). In this task, ten identical white circles with a radius of 18 pixels were displayed at random locations within a 500 × 500 pixel black square in the centre of the screen. At the beginning of each trial, three of the circles flashed red three times to indicate they were the targets that needed to be tracked before returning to white (Fig. 2b). All circles then moved in random directions across the screen at a speed of two pixels per frame refresh for a total of 8 s. The circles continued moving in the same direction unless they hit the edge of the black square where they would then bounce in the opposite direction and continue their trajectory. After 8 s, all circles came to a complete stop and the participant had to indicate, via mouse clicks, the three target circles. When selected, a circle turned green if it was a target object, or red if it was not. After three selections, the total score of that trial was displayed (i.e., “2/3 correct.”) before moving on to the next trial. There were four practice trials followed by 25 testing trials. In the testing trials, a staircase procedure was used where the speed of the circles increased by 1 pixel per frame if all targets were correctly identified and decreased by 1 pixel per frame if all targets were not correctly identified. The testing stage terminated after six reversals or 25 trials (which ever came first). The dependent variable was the average speed of movement of the last five trials (Souza & Oberauer, 2017; Zwierko et al., 2022).

##### Brief psychomotor vigilance task (PVT-B)

The PVT-B is a validated 3-min version of the PVT requiring participants to sustain attention and react as quickly as possible to stimuli presented at random intervals (Basner et al., 2011). The PVT-B involves sustained attention, processing speed, and inhibitory control. Participants were shown a red traffic light and instructed to left-click the mouse as soon as the traffic light changed to green. The delay between the light changing green and the mouse click was recorded as the participant’s RT and was displayed after each mouse click (Fig. 2c). Each participant completed an average of 58 (*SD* = 3.16) trials during the task. The dependent variable was the participants’ RT averaged over the entire session. Trials with a RT < 100 ms were not included in each participant’s mean RT (Basner et al., 2011). Although some studies use the reciprocal RT due to its sensitivity to total and partial sleep loss (Basner & Dinges, 2011), sleep deprivation was neither controlled nor a variable of interest in the present study, and thus the raw mean RT was reported for ease of interpretation.

#### Visuospatial working memory tasks

Three tasks were used to assess different domains of visuospatial working memory including object working memory and spatial working memory.

##### Austin Maze (AM)

The Austin Maze is a computerised task that measures visuospatial ability and memory (Bowden, 1989, 1992; Crowe et al., 1999; Stolwyk et al., 2013). Subjects plotted a course through a chequerboard maze by selecting black squares from a green starting block and identifying the correct path to the green finish block through trial-and-error (Fig. 2d). Each time a square was selected via mouse click, it would turn green if it was the correct path and flash red if it was not the correct path. The current study allowed for one practice trial, followed by a total of ten trials. The dependent variables were the total number of errors made, and the total time taken in seconds over the ten test trials. Although some studies may utilise ‘errors to criterion’ (i.e., three error-free trials), results show that there is a strong correlation between cumulative number of errors within the first ten trials and cumulative errors to criterion (Bowden et al., 1992).

##### Object 2back task (O2B)

This task measures object working memory and uses a library of 30 novel abstract shapes which are based on previous designs (Mueller, 2010; Caminiti et al., 2015; Chun & Jiang, 1999; Fiser & Aslin, 2001). Participants were presented one shape at a time with a stimulus duration of 2 s and an inter-stimulus interval of 2 s in which a blank screen was presented. Participants were instructed to press the ‘F’ key if the image shown matched the image shown two trials previously and the ‘J’ key if it did not match the image shown two trials previously (Fig. 2e). Trials where no response was made (i.e., timed out) were scored as incorrect. The task began with ten practice trials with on-screen instructions and feedback of accuracy. In the testing stage, participants were shown 60 presentations in two blocks of 30 with a break in between. Each block contained eight target trials where the image matched the image shown two trials previously and six distractor trials where the image matched the image shown one trial previously. These trials and images were presented in an order that ensured no two blocks were alike. The dependent variable was the percentage of correct trials after the 60 presentations as utilised by Caminiti et al. (2015).

##### Computerised spatial span test (C-SST)

A version of the Corsi Block-Tapping Test (Corsi, 1973) modified for computer was used to assess visuospatial working memory (Woods et al., 2016). In this task, ten white squares (90 × 90 pixels) were displayed within a 1,400 × 800 pixel black canvas in the centre of the screen. Five squares were displayed in each hemifield within a 5 × 5 grid (grid lines not visible to participants), with only one square in each horizontal row and vertical column. Random horizontal and vertical offsets prevented squares from being perfectly aligned (Fig. 2f). At the beginning of each trial, one square at a time would flash green for 500 ms before returning to white. Squares only flashed green once per trial. Once the squares had stopped flashing, the on-screen instructions instructed participants to select squares in the same order as they were presented, or in the reverse order they were presented. Once a square was selected, it turned green and could not be selected again. After the total number of squares were clicked corresponding to the number of squares that were flashed green, the total score for that trial was displayed (e.g., “3/4 correct”) before continuing to the next trial. If the sequence was clicked in the correct order, the total number of squares that flashed on the next trial was increased by 1. If there were two consecutive trials where the sequence order was not replicated, the total number of squares that were flashed on the next trial decreased by 1. The task began with four practice trials showing only six blocks (three per hemifield) beginning at two blocks that needed to be recalled, followed by 14 trials of ten blocks (five per hemifield) beginning at three blocks that needed to be recalled. Initially participants were instructed to replicate the sequence in the same order. The task was then repeated with the same four practice trials and 14 testing trials described except this time participants were instructed to replicate the sequence in the reverse order. The dependent variables were the maximum span correctly remembered for both the forward and backward versions of the task. Whilst various scoring metrics are available for this task (see Woods et al., 2016), the maximum sequence length correctly reproduced (aka Corsi Span) was used as this is the most reported measure for studies using this task to assess spatial memory (Arce & McMullen, 2021).

#### Cognitive flexibility task

##### Wisconsin Card Sorting Test

The Wisconsin Card Sorting Test (WCST; Berg, 1948) 64-card version was used to measure cognitive flexibility. In this task, four unchanging stimulus cards were presented at the top of the screen that varied based on three dimensions: shape, colour, and number. Below these cards, one matching card was shown that the participant had to match to one of the stimulus cards based on an unknown sorting rule. For example, if a card with two green circles was displayed and the matching rule was colour, the participant would select the stimulus card displaying a green shape (regardless of whether the shape and number matched the matching card; Fig. 2g). Participants were not told the sorting rule and had to learn the matching rule through trial and error. Feedback was shown as to whether they were correct via a green tick or incorrect via a red cross. After ten cards had correctly been sorted, the matching rule changed without warning (e.g., from colour to shape) and the participant had to learn the new matching rule. The task ended after 64 cards had been matched or six categories had been completed. The dependent variables were the number of perseverative errors made (i.e., sorting on an incorrect matching rule despite feedback advising it was incorrect), the number of correctly sorted cards, and the number of correctly sorted sets. See Miles et al. (2021) for more information regarding scoring of the WCST.

#### Top-down vs. bottom-up attention task

##### Visual attentional capture and control task (VACCT)

This novel visual search task is designed to capture individual differences in top-down and bottom-up processing in which the observer is required to search for a target shape amongst a matrix of its paired distractor shape. Shape pairs were ‘T’ and ‘L’, ‘E’ and ‘F’, ‘2’ and ‘5’, with either member of the pair able to be the target. Each trial began with a 120 × 120 pixel fixation cross displayed in the centre of an 800 × 600 pixel canvas for 1 s. A total of 40 shapes (39 distractors of the same shape and 1 target shape) were then displayed, arranged in an 8 × 5 grid. Each shape was displayed in its normal orientation (i.e., L), or rotated by 180 degrees (i.e., ⅂; Fig. 2h). Each shape was 100 pixels in height and 60 or 80 pixels in width. The position of the target within the array was randomised, except that the target was never displayed in the middle of the canvas (i.e., to the immediate left or right of the fixation cross). Once the participant had identified the target shape, they were instructed to click on it with the mouse as quickly as possible. Their RT was recorded as the duration between the onset of stimulus presentation and the mouse click (in ms). There was no limit to the search duration, and each trial ended once the target had been successfully identified. To reduce the disproportional influence of trials of extended search time, whilst not removing overall individual variability, Winsorisation was applied to the data for each participant by replacing trials above their individual 90th percentile with the value at the 90th percentile across all their trials for each condition. The task had two conditions: a dynamic condition where the target jittered in a diagonal fashion (i.e., left and up by 2 pixels then down and right by 2 pixels) at intervals of 50 ms to produce a bottom-up ‘pop out’ effect whereby attention was reflexively drawn to the target, and a static condition where the target was stationary, placing a reliance on top-down processes to identify the target (Niedeggen et al., 2001).

#### Processing speed task

##### Subtle cognitive impairment Test (SCIT)

The SCIT is a computer-based visual discrimination task that is particularly sensitive to processing speed and accuracy of decision making (Bruce et al., 2014; Friedman et al., 2011). The letter ‘H’ is briefly displayed on the screen with one side being shorter than the other (either top or bottom portion). The participant indicates which side is shorter by pressing a corresponding button on the keyboard. The ‘H’ is displayed for varying durations. The shortest durations (16–64 ms), referred to as the ‘head’ of the curve, are available for subconscious evaluation. Longer durations (80–128 ms), referred to as the ‘tail’ of the curve, are available for conscious evaluation (Fig. 2i). The computer records RTs (to measure processing speed) and error rates (to measure decision efficacy) for both the head and tail. The task begins with a 4-min practice period designed to familiarise participants with the test and the varying exposure durations, followed by 64 testing trials lasting approximately 4 min. The dependent variables are:

1. Percentage of errors for the head of the curve (SCIT-E_H_);
2. Percentage of errors for the tail of the curve (SCIT-E_T_);
3. Mean RT for the head of the curve (SCIT-RT_H_);
4. Mean RT for the tail of the curve (SCIT-RT_T_) as described by (Bruce et al., 2014).

### Procedure

Ethics approval was granted by RMIT University’s Human Ethics Committee (HEC #2024–24795-24910). Participants were recruited from the university via online announcements and flyers posted around university campuses, and snowball sampling. The AFFT, Austin Maze, Brief Psychomotor Vigilance Task, Subtle Cognitive Impairment Test, and Visual Attentional Capture and Control Task were all completed as part of a larger research project for which participants were reimbursed AUD$25. The only tasks from the previously completed project not included in analyses for this study were a personality inventory, a gaming experience questionnaire, and a non-validated privately owned visual search task, as these did not align with the scope of the present study. Performance on these tasks was not used to inform the recruitment procedure. No data from this study have been reported or published elsewhere. Of the executive function battery, the complete set of data is reported. Participants who had completed all tasks in the previous project and indicated they wished to be contacted about future studies were contacted to participate in a follow-up study consisting of the Flanker, Multiple Object Tracking, Object 2back Task, Computerised Spatial Span Task, and Wisconsin Card Sorting Test for an additional reimbursement of AUD$50. This battery of testing was completed online via the participants’ personal computer or laptop. Participants were instructed to complete the experiment in an environment free of distraction and to ensure they completed the experiment to the best of their ability, as scores below minimal performance thresholds would be excluded from the experiment, and they would receive no reimbursement.

This study was not preregistered. Although previously published scoring and cutoff procedures were applied to the tasks used, different outcome metrics could have influenced the results. To test this possibility, we also ran our analyses using various other approaches to defining the variable of interest. In each case, the substantial pattern of results was unchanged. Nevertheless, the data are openly available for researchers to explore alternative operationalisations of the outcome measures used (see Online Supplementary Materials).

### Data cleaning

The data were cleaned using code written in Python version 3.12, and data analyses were conducted using jamovi version 2.3.28.0 (The jamovi project, 2025). Nine participants had their data for individual tasks manually removed due to experiencing technical difficulties during the task (e.g., browser needing to be refreshed resulting in a task having to be restarted or failing to understand the task instructions). This resulted in two different participants being removed for the Multiple Object Tracking and Wisconsin Card Sorting Test, and one participant was removed from the Austin Maze, Subtle Cognitive Impairment Test, Flanker, Visual Attentional Capture and Control Task (static condition only), and Computerised Spatial Span Task (backward version only).

The data were then checked for outliers using a two-pass procedure where scores at the subject level that were 3.5 *SD*s beyond the sample mean for each task were removed on each pass (Burgoyne et al., 2023). This process removed < 1% of all data. Participants’ data for the remaining tasks were retained for correlation analyses but were excluded listwise for the factor analysis.

## Results

### Descriptive statistics

Descriptive statistics for all outcome variables are shown in Table 1. Most variables demonstrated no significant deviation from normality. All variables demonstrated an adequate spread of scores, confirming their suitability for correlational analyses. The intercorrelations between all outcome variables are shown in Table 2. Specific to AFFT scores, the data revealed significant weak correlations with the static RT on the Visual Attentional Capture and Control task, errors and time taken on the Austin Maze, error rate on the head of the Subtle Cognitive Impairment Test, and total score on the Object 2back task.

**Table 1:** Descriptive statistics for all outcome variables

Variable	*N*	Mean (*SD*)	Range	Skewness	Kurtosis	Reliability
AFFT Score	260	8.26 (2.96)	1.00–17.00	0.15	0.04	Set A =.40 Set B =.60
VACCT Dynamic RT (ms)	210	870 (172)	486–1418	0.79	0.74	α =.96
VACCT Static RT (ms)	206	3,621 (948)	1,947–6,895	1.03	0.85	α =.86
PVT-B Mean RT (ms)	258	290 (38)	201–425	0.51	0.62	α =.96
AM Errors	256	39.64 (21.57)	7.00–112.00	0.85	0.26	*r* =.95
AM Total Time Taken	259	257 (75)	120–494	0.86	0.53	*r* =.93
SCIT-RT_H_ (ms)	259	642 (100)	383–925	0.08	−0.13	α =.93
SCIT-E_H_ (%)	258	28.53 (14.40)	0.00–78.00	0.49	0.49	α =.76
SCIT-RT_T_ (ms)	258	518 (76)	373–742	0.48	−0.26	α =.95
SCIT-E_T_ (%)	250	2.68 (3.79)	0.00–19.00	1.84	3.79	α =.86
Flanker Deadline	220	532 (79)	380–920	1.36	3.26	N/A
MOT Average Speed	256	3.75 (1.57)	0.40–9.00	0.49	0.00	N/A
C-SST Forward Span	260	6.86 (0.95)	5.00–9.00	0.17	−0.26	N/A
C-SST Backward Span	259	6.44 (1.10)	4.00–9.00	0.15	−0.38	N/A
O2B Total	259	74.62 (15.60)	22.41–98.28	−1.11	1.02	α =.89
WCST Perseverative Errors	254	9.80 (5.91)	1.00–28.00	0.9	0.01	ICC =.72
WCST Sets Sorted	258	4.33 (1.11)	1.00–6.00	−0.34	−0.14	ICC =.84
WCST Correct	257	48.50 (6.34)	28.00–58.00	−1.07	0.76	*r* =.90

AFFT = Alternate Forms Flicker Task, VACCT = Visual Attentional Capture and Control Task, PVT-B = Brief Psychomotor Vigilance Task Mean Reaction Time, AM = Austin Maze, SCIT = Subtle Cognitive Impairment Test, RT_H_ = Response Time Head, E_H_ = Error Rate Head, RT_T_ = Response Time Tail, E_T_ = Error Rate Tail, Flanker Deadline = Final Duration to Respond, MOT = Multiple Objective Tracking, C-SST = Computerised Spatial Span Test, O2B Total = Object 2back % of Trials Correct, WCST = Wisconsin Card Sorting Test

The VACCT was a task introduced later into the original research project; the smaller sample size is due to all participants not completing this task and not due to outlier removal or manual exclusion. AFFT and AM reliability based on split-half coefficients with Spearman Brown correction. PVT-B Mean RT Cronbach’s Alpha based on the first valid 60 trials. Internal consistency was not calculated for adaptive and deadline variables due to the nature of these tasks (Draheim et al., 2022). WCST Perseverative Errors and Sets Sorted based on Intraclass Correlation Coefficients by Chiu and Lee (2021)

**Table 2:** Intercorrelations between outcome variables

	1	2	3	4	5	6	7	8	9	10	11	12	13	14	15	16	17
1. AFFT Score	-																
2. VACCT Dynamic RT	-.06	-															
3. VACCT Static RT	-.33**	.25**	-														
4. PVT Mean RT	-.10	.40**	.19**	-													
5. AM Errors	-.26**	.05	.22**	-.02	-												
6. AM Total Time Taken	-.15*	.38**	.28**	.26**	.30**	-											
7. SCIT-RT_H_	-.10	.19**	.21**	.18**	.08	.26**	-										
8. SCIT-E_H_	-.18**	.21**	.17*	.29**	.19**	.31**	.65**	-									
9. SCIT-RT_T_	-.15*	.27**	.15*	.18**	.13*	.26**	.78**	.48**	-								
10. SCIT-E_T_	-.14*	.16*	.22**	.10	.30**	.22**	.23**	.40**	.41**	-							
11. Flanker Deadline	-.06	.17*	.13	.23**	.04	.24**	.08	.14*	.15*	.25**	-						
12. MOT Average Speed	.15*	.01	-.19**	.04	-.28**	-.14*	-.07	-.14*	-.15*	-.20**	-.15*	-					
13. C-SST Forward Span	.11	-.05	-.26**	.00	-.23**	-.23**	-.14*	-.11	-.17**	-.19**	-.11	.17**	-				
14. C-SST Backward Span	.12	.01	-.30**	-.06	-.30**	-.26**	-.11	-.08	-.13*	-.22**	-.23**	.25**	.47**	-			
15. O2B Total	.17**	-.05	-.23**	-.09	-.33**	-.18**	-.11	-.27**	-.15*	-.26**	-.22**	.35**	.22**	.32**	-		
16. WCST Perseverative Errors	-.11	.06	.16*	.03	.32**	.20**	.11	.12	.14*	.17**	.11	-.15*	-.13*	-.16**	-.29**	-	
17. WCST Sets Sorted	.04	-.02	-.15*	-.03	-.26**	-.15*	-.05	-.07	-.09	-.19**	-.16*	.13*	.09	.06	.21**	-.71**	-
18. WCST Correct	.08	-.08	-.19**	-.07	-.31**	-.20**	-.13*	-.15*	-.15*	-.23**	-.14*	.12	.13*	.15*	.23**	-.87**	.78**

** p <.01. * *p* <.05. AFFT = Alternate Forms Flicker Task, VACCT = Visual Attentional Capture and Control Task, PVT-B = Brief Psychomotor Vigilance Task Mean Reaction Time, AM = Austin Maze, SCIT = Subtle Cognitive Impairment Test, RT_H_ = Response Time Head, E_H_ = Error Rate Head, RT_T_ = Response Time Tail, E_T_ = Error Rate Tail, Flanker Deadline = Final Duration to Respond, MOT = Multiple Objective Tracking, C-SST = Computerised Spatial Span Test, O2B Total = Object 2back % of Trials Correct, WCST = Wisconsin Card Sorting Test

### Factor analysis

An exploratory factor analysis using Principal Axis Factoring extraction with Promax rotation examined the tasks’ latent structure. Table 3 shows three factors were extracted accounting for 40% of the variance; Factor 1 (Cognitive Flexibility), Factor 2 (Visuospatial Working Memory), and Factor 3 (Attention and Processing Speed). Although there was a theoretical expectation that three factors would emerge, alternative factor solutions were explored (see Online Supplementary Materials). The three-factor solution was retained as it represented the most parsimonious structure with each factor having a minimum of three variables and minimal cross loading (Costello & Osborne, 2005; see Appendix A for full details).

**Table 3:** Pattern matrix obtained via principal axis factoring with promax rotation

Variable	Factor 1: Cognitive Flexibility	Factor 2: Visuospatial Working Memory	Factor 3: Attention and Processing Speed
WCST Correct	**.97**	-.06	.06
WCST Perseverative Errors	**.90**	.04	.00
WCST Sets Sorted	**.80**	-.08	-.02
C-SST Backward Span	-.10	**.73**	-.12
C-SST Forward Span	-.11	**.51**	.00
O2B Total	.10	**.50**	.06
VACCT Static RT	-.03	**.41**	.20
AM Errors	.19	**.41**	-.08
MOT Average Speed	.00	**.39**	-.02
SCIT-E_H_	.06	.03	**.61**
SCIT-RT_T_	.06	.04	**.55**
VACCT Dynamic RT	-.04	-.07	**.53**
PVT-B Mean RT	-.03	-.03	**.51**
Eigenvalues	2.44	1.50	1.30

Response Time and Error variables were reversed so that higher values indicate better performance. WCST = Wisconsin Card Sorting Test, C-SST Backward Span = Computerised Spatial Span Test Maximum Span, C-SST Forward Span = Computerised Spatial Span Test Maximum Span, O2B Total = Object 2back Task % of Trials Correct, VACCT = Visual Attentional Capture and Control Task, AM = Austin Maze, MOT = Multiple Objective Tracking, SCIT = Subtle Cognitive Impairment Test, E_H_ = Error Rate Head, RT_T_ = Response Time Tail, PVT-B = Brief Psychomotor Vigilance Task Mean Reaction Time

### Multiple linear regression

From these three factors, regression scores were calculated to reflect the overall ability of each participant (*n* = 190) for the cognitive processes of cognitive flexibility, visuospatial working memory, and attention and processing speed (Distefano et al., 2009). Table 4 reveals a significant weak positive correlation between visuospatial working memory performance and AFFT scores (*r* =.30), and a significant weak positive correlation between attention and processing speed performance and AFFT scores (*r* =.22). There was no significant correlation between cognitive flexibility performance and AFFT scores.

**Table 4:** Intercorrelations between change detection score and executive function performance

Variable	1.	2.	3.
1. AFFT Score	-		
2. Cognitive Flexibility	.10	-	
3. Visuospatial Working Memory	.30**	.38**	-
4. Attention and Processing Speed	.22*	.20**	.34**

** *p* <.001. * *p* <.01. AFFT = Alternate Forms Flicker Task

A multiple linear regression model using the calculated factor scores for cognitive flexibility, visuospatial working memory, and attention and processing speed were used to predict AFFT scores. Together, these variables significantly accounted for 10.0% of the variability in AFFT scores, *R* =.32. Only visuospatial working memory performance was a significant predictor of AFFT scores (Table 5).

**Table 5:** Multiple linear regression coefficients predicting alternate forms flicker task scores from cognitive flexibility, visuospatial working memory, and attention and processing speed

Variable	B	95% CI	β	t	p
Constant	8.29	[7.89, 8.69]		41.03	<.001
Cognitive Flexibility	−0.09	[−0.54, 0.35]	−0.03	−0.40	.67
Visuospatial Working Memory	0.92	[0.38, 1.45]	0.27	3.38	<.001
Attention and Processing Speed	0.47	[−0.05, 0.99]	0.13	1.78	.08

Although other studies using the CB flicker paradigm also examine RT as a performance metric, the AFFT was developed with the intention to use scores as the main dependent variable because accuracy-based measures tend to improve reliability and correlations with other cognitive tasks (Draheim et al., 2019, 2021; Snijder et al., 2024). Analysis of executive functioning performance using RT on the AFFT is reported in Appendix B for completeness but should be interpreted cautiously given the participants’ instruction to prioritise accuracy over speed.

## Discussion

The aim of the present study was to explore the role of executive functioning in CD accuracy from the perspective of individual differences. The hypothesis that tasks addressing executive functions of attention control, cognitive flexibility, visuospatial working memory, and processing speed would significantly predict CD accuracy was partially supported; with the visuospatial working memory factor being a significant predictor of AFFT scores. While neither cognitive flexibility nor attention and processing speed significantly predicted AFFT scores by themselves, they did contribute to a three-factor model that accounted for a significant amount of variance in AFFT scores. These results support previous research that has highlighted the contribution of these cognitive processes to successful CD (Andermane et al., 2019; Asano et al., 2008; Batchelder et al., 2003; Colflesh & Wiley, 2013; Huang et al., 2012; Kerrigan et al., 2017; Rizzo et al., 2009). The present results extend this understanding by demonstrating the role of individual differences in each of these cognitive processes in a single study in a large healthy adult sample.

The results of the multiple linear regression revealed that the visuospatial working memory task factor provided the most significant contribution to AFFT scores. Participants with superior visuospatial working memory ability as measured by errors on the Austin Maze, the average tracking speed on the Multiple Object Tracking task, total score on the Object 2back Task, both forward and backward span on the Computerised Spatial Span Task, as well as the average RT for the static condition of the Visual Attentional Capture and Control Task, correctly identified more changes on the AFFT.

The significant relationship between visuospatial working memory performance and AFFT scores is consistent with previous research (Huang et al., 2012; Kerrigan et al., 2017; Rizzo et al., 2009; Tseng et al., 2010). For most of the tasks loading on the visuospatial working memory factor, participants are required to maintain and update information in working memory depending on the specific task. Superior visuospatial working memory ability likely indicates an ability to maintain and update multiple objects/areas in working memory within the changing stimuli, increasing the probability of detecting a change.

Previous research has shown an involvement of attentional processes (e.g., focussed or selective attention) in successful CD (Andermane et al., 2019; Asano et al., 2008; Huang et al., 2012; Pringle et al., 2001; Simon & Rensink, 2005; Rensink et al., 1997; Rizzo et al., 2009). Whilst it was expected that an attention control factor would emerge, the present results revealed the Flanker Deadline task did not load above .3 on any of the factors in the exploratory factor analysis model. Although this outcome was unexpected, it is consistent with subsequent papers by both Draheim et al. (2023) and Burgoyne et al. (2023) that reported weak intercorrelations between the Flanker Deadline and other tasks of attention control, resulting in it being dropped from their analyses.

Whilst the results of the multiple linear regression indicated the attention and processing speed factor (consisting of the Subtle Cognitive Impairment Test (SCIT), Brief Psychomotor Vigilance Task, and Visual Attentional Capture and Control Task dynamic RT) had a weak effect size on AFFT scores, this factor was not a significant predictor. This weak effect on AFFT scores is likely driven by performance on the SCIT. The SCIT predominantly measures processing speed and efficiency (Bruce et al., 2014), with the head component also measuring pre-attentive processing, as these shorter stimulus presentations (16–64 ms) are processed with minimal conscious awareness (Forster & Davis, 1984; Mattingley et al., 2001; Bruce et al., 2014). Additionally, the error rate of the head of the SCIT is a measure of decision-making efficacy (Bruce et al., 2014), so superior performance on this component indicates an ability to make rapid and correct decisions about transiently presented stimuli. Although the presentation duration of the stimuli differs, both the SCIT (Stimulus = 16–128 ms, Mask = 1,000 ms) and AFFT (Images = 600 ms, Mask = 100 ms) involve the observation of briefly presented visual stimuli followed by a visual mask that is designed to provide visual distraction and block out visual after-images of the stimulus. Attentional capture is exemplified by poor disengagement from the CB mask (Andermane et al., 2019). Given that CB involves frequent storing and updating of scene representations (Jensen et al., 2011; Rensink et al., 2000; Simons & Rensink, 2005), and the relationship between processing speed and working memory capacity (Löffler et al., 2024), the present results suggest that superior processing speed and efficiency may aid participants in disengaging their attention from the visual mask and focus on the goal of the task. Future research should further investigate the role of attention and processing speed in predicting AFFT scores using tasks that involve a strong interference aspect requiring stronger attention control.

Lastly, the cognitive flexibility factor was a non-significant predictor and had the weakest overall impact in the multiple linear regression. These results suggest that superior ability to detect changes is better explained by the other executive functions, and particularly visuospatial working memory. However, it is acknowledged that the cognitive flexibility factor reflects task performance on the Wisconsin Card Sorting Task only and is unlikely to capture this executive function in its entirety (Hohl & Dolcos, 2024). Additional tests measuring cognitive flexibility such as the Trail Making Test (Army U.S., 1944), or the Intra–Extradimensional Set-Shifting Task (Cambridge Cognition, 2023) may have provided a more comprehensive measure of cognitive flexibility and added predictive power to the model. It should be noted that tasks in the executive function battery were chosen due to their quick administration time and suitability for online testing. Inclusion of additional tests would increase the overall testing time and contribute to participant fatigue, which can negatively impact performance (Tu et al., 2022). Future studies should include additional tasks of cognitive flexibility and/or fluid intelligence to get a more thorough understanding of this relationship between CD scores.

The present results demonstrate that the AFFT accuracy involves top-down attentional processes that engage visuospatial working memory, and to some extent, rapid cognitive processing and decision-making processes. The correlations between AFFT scores and the visuospatial working memory, and attention and processing speed factors are relatively moderate and within the range typically observed in the context of individual differences research (Gignac & Szodorai, 2016). Further, these components collectively accounted for 10% of the variance in scores on the AFFT, which represents a typical significant regression effect size in behavioural and social sciences (Ozili, 2023). However, this outcome also indicates that other, as-yet-unidentified, parameters could be responsible for the bulk of the variance. One likely parameter is the visual search strategy employed by the participants (Boot et al., 2009). Although search strategy was not tracked in the present study, it seems likely that those who adopted a search strategy allowing them to identify the Visual Attentional Capture and Control Task target quicker, were also able to identify more changes in the AFFT. Future research may wish to explore this further by administering both tasks while utilising eye-tracking hardware.

### Strengths and limitations of the present study

The advantages of the present study are:

1. The large sample size and inclusion of tasks that involve each of the core executive functions and processing speed in a single study.
2. The AFFT used in the present study has a large spread of scores, *M* = 8.26 (43.48%), *SD* = 2.96 (15.59%), range = 1–17 (5.26–89.47%), representing sufficient difficulty and discriminatory power (Draheim et al., 2022; Zorowitz & Niv, 2023), allowing for correlational analyses to explore the relationship between AFFT scores and cognitive performance.

Thus, the present study is one of the few to examine the role of individual differences in CD accuracy in healthy younger adults.

(3) Compared to previous CD studies, the present study used a wider range and variety of tests of executive function.

Knowing more about the individual differences that contribute to CB could allow for better predictions about what changes are difficult to detect, and how CB could be controlled or prevented in the external world (Schankin et al., 2017).

A potential limitation of the present study is that CD performance was measured with a single CD task. Although this is standard practice, scores on the AFFT reflects both construct-relevant and task-specific variance. Future research could incorporate additional tasks of CD where the shared variance of these tasks can be extracted, providing a more comprehensive measure of CD ability.

Additionally, the relationships between tasks at the factor level and with AFFT scores may have been stronger if the demands of each of the executive functions was manipulated at the trial level resulting in varying difficulty of the task. Further, accounting for trial-by-trial variability may help with attenuated correlations in studies of individual differences (Rouder & Haaf, 2019). The present study utilised commonly used tasks and outcome measures to assess each of the executive functions;0F^1^ however, recent studies have shown modifications to tasks measuring executive function can lead to more reliable and valid measures of individual differences (Burgoyne et al., 2023; Draheim et al., 2022, 2023; Löffler et al., 2024). Future research could build on the present study with such tasks to determine whether the relationships between AFFT accuracy and executive functioning are strengthened.

### Implications

The findings of the present study indicate that individual differences in visuospatial working memory ability underpin accuracy on the AFFT. This finding has important implications for why the phenomenon of CB exists and why some individuals are better than others at identifying changes. These insights could have practical benefits, such as identifying drivers who may have a greater risk of serious car crashes due to failing to detect hazards on the road (Beanland et al., 2017).

Previous research has used artificial display versions of the one-shot CD paradigm (Johnson et al., 2013; Rouder et al., 2011; Vogel et al., 2001; Vogel & Machizawa, 2004) and the flicker paradigm (Pailian et al., 2020) to estimate working memory capacity. Thus, the association between AFFT scores and visuospatial working memory is consistent with previous studies. The present study helps confirm that performance on a flicker task using naturalistic imagery utilises similar cognitive mechanisms to CD using artificial displays.

However, the results of the present study show that only a portion of the variance in accuracy on the AFFT could be attributed to visuospatial working memory performance. Thus, whilst visuospatial working memory ability plays a role in successful detection of changes in naturalistic imagery, other cognitive processes such as search strategy and attention control may explain more of the variance than visual working memory capacity alone.

Nevertheless, if visuospatial working memory ability plays a key role in CD accuracy, the AFFT could be used to inform and improve the visual search abilities of occupations where visuospatial skills are necessary, such as radiology (Birchall, 2015), potentially reducing diagnostic error rates (Waite et al., 2020).

## Summary and conclusions

The present study explored the relationship between executive functioning, processing speed, and accuracy on a CD flicker task suited to individual differences. The findings are consistent with previous research looking at group comparisons but extend our understanding by showing that individuals with superior visuospatial working memory ability, and to some extent, more rapid processing speed and efficacy tend to detect more changes between alternating visual stimuli. Further research is required to understand the role of other cognitive processes, including search strategy and sub-components of executive function.

## Supplementary Information

Below is the link to the electronic supplementary material.

Supplementary file1 (DOCX 495 KB)

## Data Availability

The material used and data supporting the findings of this study are available at: 10.25439/rmt.30856451

## Footnotes

1 Although the Visual Attentional Capture and Control Task is novel, this task was designed to maximise between-subject variance and was the strongest predictor with scores on the Alternate Forms Flicker Task.

## Code availability

The Alternate Forms Flicker Task used on Gorilla online in this experiment can be accessed at: https://app.gorilla.sc/openmaterials/1172473

## References

[CR1] Andermane, N., Bosten, J. M., Seth, A. K., & Ward, J. (2019). Individual differences in change blindness are predicted by the strength and stability of visual representations. *Neuroscience of Consciousness*, *2019*(1). 10.1093/nc/niy010 10.1093/nc/niy010 PMC6345093 30697440

[CR2] Arce, T., & McMullen, K. (2021). The corsi block-tapping test: Evaluating methodological practices with an eye towards modern digital frameworks. *Computers in Human Behavior Reports*. 10.1016/j.chbr.2021.100099

[CR3] Army U. S. (1944). *Army individual test battery*. Manual of Directions and Scoring.

[CR4] Asano, M., Kanaya, S., & Yokosawa, K. (2008). Proofreaders show a generalized ability to allocate spatial attention to detect changes. *Psychologia,**51*(2), 126–141. 10.2117/psysoc.2008.126

[CR5] Ashwin, C., Wheelwright, S., & Baron-Cohen, S. (2017). Differences in change blindness to real-life scenes in adults with autism spectrum conditions. *PLoS ONE*. 10.1371/journal.pone.0185120 29020056 10.1371/journal.pone.0185120 PMC5636097

[CR6] Basner, M., & Dinges, D. F. (2011). Maximizing sensitivity of the psychomotor vigilance test (PVT) to sleep loss. *Sleep,**34*(5), 581–591. 10.1093/sleep/34.5.581 21532951 10.1093/sleep/34.5.581 PMC3079937

[CR7] Basner, M., Mollicone, D., & Dinges, D. F. (2011). Validity and sensitivity of a brief psychomotor vigilance test (PVT-B) to total and partial sleep deprivation. *Acta Astronautica,**69*(11–12), 949–959. 10.1016/j.actaastro.2011.07.015 22025811 10.1016/j.actaastro.2011.07.015 PMC3197786

[CR8] Batchelder, S., Rizzo, M., Vanderleest, R., & Vecera, S. (2003). Traffic Scene Related Change Blindness in Older Drivers. *Driving Assessment Conference,**2*(2003), 177–181. 10.17077/drivingassessment.1117

[CR9] Beanland, V., Filtness, A. J., & Jeans, R. (2017). Change detection in urban and rural driving scenes: Effects of target type and safety relevance on change blindness. *Accident Analysis and Prevention*, *100*, 111–122. 10.1016/j.aap.2017.01.011 10.1016/j.aap.2017.01.011 28130981

[CR10] Berg, E. A. (1948). A simple objective technique for measuring flexibility in thinking. *The Journal of General Psychology,**39*(1), 15–22. 10.1080/00221309.1948.9918159 18889466 10.1080/00221309.1948.9918159

[CR11] Birchall, D. (2015). Spatial ability in radiologists: A necessary prerequisite? *The British Journal of Radiology,**88*(1049), Article 20140511. 10.1259/bjr.20140511 25756868 10.1259/bjr.20140511 PMC4628467

[CR12] Boot, W. R., Becic, E., & Kramer, A. F. (2009). Stable individual differences in search strategy?: The effect of task demands and motivational factors on scanning strategy in visual search. *Journal of Vision,**9*(3), 7–7. 10.1167/9.3.7 19757946 10.1167/9.3.7

[CR13] Bowden, S. (1989). Maze learning: Reliability and equivalence of alternate pathways. *Clinical Neuropsychologist*, *3*(2), 137–144. 10.1080/13854048908403286

[CR14] Bowden, S., Dumendzic, J., Hopper, J., Kinsella, G., Clifford, C., & Tucker, A. (1992). Healthy adults’ performance on the austin maze. *Clinical Neuropsychologist,**6*(1), 43–52. 10.1080/13854049208404116

[CR15] Bruce, K., Robinson, S., Smith, J., & Yelland, G. (2014). Validity of a screening tool for detecting subtle cognitive impairment in the middle-aged and elderly. *Clinical Interventions in Aging*, 2165–2176. 10.2147/CIA.S68363 10.2147/CIA.S68363 PMC4270303 25540581

[CR16] Brysbaert, M. (2024). Designing and evaluating tasks to measure individual differences in experimental psychology: A tutorial. *Cognitive Research: Principles and Implications,**9*(1), 11. 10.1186/s41235-024-00540-2 38411837 10.1186/s41235-024-00540-2 PMC10899130

[CR17] Burgoyne, A. P., Tsukahara, J. S., Mashburn, C. A., Pak, R., & Engle, R. W. (2023). Nature and measurement of attention control. *Journal of Experimental Psychology: General,**152*(8), 2369–2402. 10.1037/xge0001408 37079831 10.1037/xge0001408

[CR18] Cambridge Cognition. (2023). *Intra-Extra Dimensional Set Shift (IED)*. Cambridge Cognition Ltd. https://cambridgecognition.com/intra-extra-dimensional-set-shift-ied/

[CR19] Caminiti, S. P., Siri, C., Guidi, L., Antonini, A., & Perani, D. (2015). The neural correlates of spatial and object working memory in elderly and Parkinson’s disease subjects. *Behavioural Neurology*. 10.1155/2015/123636 25861157 10.1155/2015/123636 PMC4378329

[CR20] Caplovitz, G. P., Fendrich, R., & Hughes, H. C. (2008). Failures to see: Attentive blank stares revealed by change blindness. *Consciousness and Cognition,**17*(3), 877–886. 10.1016/j.concog.2007.08.006 17931887 10.1016/j.concog.2007.08.006

[CR21] Cepeda, N. J., Blackwell, K. A., & Munakata, Y. (2013). Speed isn’t everything: Complex processing speed measures mask individual differences and developmental changes in executive control. *Developmental Science,**16*(2), 269–286. 10.1111/desc.12024 23432836 10.1111/desc.12024 PMC3582037

[CR22] Chiu, E. C., & Lee, S. C. (2021). Test–retest reliability of the Wisconsin Card Sorting Test in people with schizophrenia. *Disability and Rehabilitation,**43*(7), 996–1000. 10.1080/09638288.2019.1647295 31361972 10.1080/09638288.2019.1647295

[CR23] Chun, M. M., & Jiang, Y. (1999). Top-down attentional guidance based on implicit learning of visual covariation. *Psychological Science,**10*(4), 360–365. 10.1111/1467-9280.00168

[CR24] Cohen, M. A., Cavanagh, P., Chun, M. M., & Nakayama, K. (2012). The attentional requirements of consciousness. *Trends in Cognitive Sciences,**16*(8), 411–417. 10.1016/j.tics.2012.06.013 22795561 10.1016/j.tics.2012.06.013

[CR25] Colflesh, G. J. H., & Wiley, J. (2013). Drunk, but not blind: The effects of alcohol intoxication on change blindness. *Consciousness and Cognition,**22*(1), 231–236. 10.1016/j.concog.2013.01.001 23357240 10.1016/j.concog.2013.01.001

[CR26] Colzato, L. S., Van Wouwe, N. C., Lavender, T. J., & Hommel, B. (2006). Intelligence and cognitive flexibility: Fluid intelligence correlates with feature “unbinding” across perception and action. *Psychonomic Bulletin & Review,**13*(6), 1043–1048. 10.3758/bf03213923 17484433 10.3758/bf03213923

[CR27] Corsi, P. M. (1973). Human memory and the medial temporal region of the brain. *Dissertation Abstracts International,**34*(2-B), 891.

[CR28] Costello, A. B., & Osborne, J. (2005). Best practices in exploratory factor analysis: Four recommendations for getting the most from your analysis. *Practical Assessment, Research, and Evaluation,**10*(1), 7. 10.7275/jyj1-4868

[CR29] Crowe, S. F., Barclay, L., Brennan, S., Farkas, L., Gould, E., Katchmarsky, S., & Vayda, S. (1999). The cognitive determinants of performance on the Austin Maze. *Journal of the International Neuropsychological Society,**5*(1), 1–9. 10.1017/S1355617799511016 9989018 10.1017/s1355617799511016

[CR30] Diamond, A. (2013). Executive functions. *Annual Review of Psychology,**64*(1), 135–168. 10.1146/annurev-psych-113011-143750 23020641 10.1146/annurev-psych-113011-143750 PMC4084861

[CR31] Distefano, C., Zhu, M., & Mîndrilã, D. (2009). Understanding and using factor scores: Considerations for the applied researcher. *Practical Assessment, Research, and Evaluation,**14*(1), 20. 10.7275/da8t-4g52

[CR32] Draheim, C., Mashburn, C. A., Martin, J. D., & Engle, R. W. (2019). Reaction time in differential and developmental research: A review and commentary on the problems and alternatives. *Psychological Bulletin,**145*(5), 508–535. 10.1037/bul0000192 30896187 10.1037/bul0000192

[CR33] Draheim, C., Tsukahara, J. S., Martin, J. D., Mashburn, C. A., & Engle, R. W. (2021). A toolbox approach to improving the measurement of attention control. *Journal of Experimental Psychology: General,**150*(2), 242–275. 10.1037/xge0000783 32700925 10.1037/xge0000783

[CR34] Draheim, C., Pak, R., Draheim, A. A., & Engle, R. W. (2022). The role of attention control in complex real-world tasks. *Psychonomic Bulletin & Review,**29*(4), 1143–1197. 10.3758/s13423-021-02052-2 35167106 10.3758/s13423-021-02052-2 PMC8853083

[CR35] Draheim, C., Tshukara, J. S., & Engle, R. W. (2023). Replication and extension of the toolbox approach to measuring attention control. *Behavior Research Methods,**56*(3), 2135–2157. 10.3758/s13428-023-02140-2 37253957 10.3758/s13428-023-02140-2 PMC10228888

[CR36] Edwards, H. M., Jackson, J. G., & Evans, H. (2022). Neuroticism as a covariate of cognitive task performance in individuals with tinnitus. *Frontiers in Psychology*, *13*. 10.3389/fpsyg.2022.906476 10.3389/fpsyg.2022.906476 PMC9379139 35983209

[CR37] Eledum, H. Y. (2017). A Monte Carlo study of the effects of variability and outliers on the linear correlation coefficient. *Journal of Modern Applied Statistical Methods,**16*(2), 231–255. 10.22237/jmasm/1509495180

[CR38] Figueroa, I. J., & Youmans, R. J. (2012). Individual differences in cognitive flexibility predict performance in vigilance tasks. *Proceedings of the Human Factors and Ergonomics Society,**56*(1), 1099–1103. 10.1177/1071181312561239

[CR39] File, D., Petro, B., Gaál, Z. A., Csikós, N., & Czigler, I. (2022). Automatic change detection: Mismatch negativity and the now-classic Rensink, O’Reagan, and Clark (1997) stimuli. *Frontiers in Psychology*. 10.3389/fpsyg.2022.975714 36092095 10.3389/fpsyg.2022.975714 PMC9458516

[CR40] Fiser, J., & Aslin, R. N. (2001). Unsupervised statistical learning of higher-order spatial structures from visual scenes. *Psychological Science,**12*(6), 499–504. 10.1111/1467-9280.00392 11760138 10.1111/1467-9280.00392

[CR41] Fletcher-Watson, S., Leekam, S. R., Connolly, B., Collis, J. M., Findlay, J. M., Mcconachie, H., & Rodgers, J. (2012). Attenuation of change blindness in children with autism spectrum disorders. *British Journal of Developmental Psychology,**30*(3), 446–458. 10.1111/j.2044-835X.2011.02054.x 22882373 10.1111/j.2044-835X.2011.02054.x

[CR42] Forster, K. I., & Davis, C. (1984). Repetition priming and frequency attenuation in lexical access. *Journal of Experimental Psychology: Learning, Memory, and Cognition,**10*(4), 680–698. 10.1037/0278-7393.10.4.680

[CR43] Friedman, T. W., Robinson, S. R., & Yelland, G. W. (2011). Impaired perceptual judgment at low blood alcohol concentrations. *Alcohol,**45*(7), 711–718. 10.1016/j.alcohol.2010.10.007 21145695 10.1016/j.alcohol.2010.10.007

[CR44] Garner, L. D., & Robison, M. K. (2025). Individual differences in attention capture, control, and working memory. *Journal of Experimental Psychology: Human Perception and Performance,**51*(2), 243–259. 10.1037/xhp0001264 39913494 10.1037/xhp0001264

[CR45] Gignac, G. E., & Szodorai, E. T. (2016). Effect size guidelines for individual differences researchers. *Personality and Individual Differences,**102*, 74–78. 10.1016/j.paid.2016.06.069

[CR46] Hedge, C., Powell, G., & Sumner, P. (2018). The reliability paradox: Why robust cognitive tasks do not produce reliable individual differences. *Behavior Research Methods,**50*(3), 1166–1186. 10.3758/s13428-017-0935-1 28726177 10.3758/s13428-017-0935-1 PMC5990556

[CR47] Hohl, K., & Dolcos, S. (2024). Measuring cognitive flexibility: A brief review of neuropsychological, self-report, and neuroscientific approaches. *Frontiers in Human Neuroscience*. 10.3389/fnhum.2024.1331960 38439938 10.3389/fnhum.2024.1331960 PMC10910035

[CR48] Huang, L., Mo, L., & Li, Y. (2012). Measuring the interrelations among multiple paradigms of visual attention: An individual differences approach. *Journal of Experimental Psychology: Human Perception and Performance,**38*(2), 414–428. 10.1037/a0026314 22250865 10.1037/a0026314

[CR49] Introzzi, I., Canet-Juric, L., Montes, S., López, S., & Mascarello, G. (2015). Inhibitory processes and cognitive flexibility: evidence for the theory of attentional inertia. *International Journal of Psychological Research*, *8*(2), 61–75. 10.21500/20112084.1510

[CR50] Jensen, M. S., Yao, R., Street, W. N., & Simons, D. J. (2011). Change blindness and inattentional blindness. *Wiley Interdisciplinary Reviews: Cognitive Science,**2*(5), 529–546. 10.1002/wcs.130 26302304 10.1002/wcs.130

[CR51] Johnson, M. K., McMahon, R. P., Robinson, B. M., Harvey, A. N., Hahn, B., Leonard, C. J., Luck, S. J., & Gold, J. M. (2013). The relationship between working memory capacity and broad measures of cognitive ability in healthy adults and people with schizophrenia. *Neuropsychology,**27*(2), 220–229. 10.1037/a0032060 23527650 10.1037/a0032060 PMC3746349

[CR52] Kerrigan, L., Thomas, M. S. C., Bright, P., & Filippi, R. (2017). Evidence of an advantage in visuo-spatial memory for bilingual compared to monolingual speakers. *Bilingualism,**20*(3), 602–612. 10.1017/S1366728915000917

[CR53] Löffler, C., Frischkorn, G. T., Hagemann, D., Sadus, K., & Schubert, A. L. (2024). The common factor of executive functions measures nothing but speed of information uptake. *Psychological Research,**88*(4), 1092–1114. 10.1007/s00426-023-01924-7 38372769 10.1007/s00426-023-01924-7 PMC11143038

[CR54] Mattingley, J. B., Rich, A. N., Yelland, G., & Bradshaw, J. L. (2001). Unconscious priming eliminates automatic binding of colour and alphanumeric form in synaesthesia. *Nature,**410*(6828), 580–582. 10.1038/35069062 11279495 10.1038/35069062

[CR55] Meyerhoff, H. S., & Papenmeier, F. (2020). Individual differences in visual attention: A short, reliable, open-source, and multilingual test of multiple object tracking in PsychoPy. *Behavior Research Methods,**52*(6), 2556–2566. 10.3758/s13428-020-01413-4 32495028 10.3758/s13428-020-01413-4

[CR56] Miles, S., Howlett, C. A., Berryman, C., Nedeljkovic, M., Moseley, G. L., & Phillipou, A. (2021). Considerations for using the Wisconsin Card Sorting Test to assess cognitive flexibility. *Behavior Research Methods,**53*(5), 2083–2091. 10.3758/s13428-021-01551-3 33754321 10.3758/s13428-021-01551-3

[CR57] Mueller, S. (2010, August 15). *Attneave shapes*. PEBL Blog. http://peblblog.blogspot.com/2010/08/attneave-shapes.html

[CR58] Mundfrom, D. J., Shaw, D. G., & Ke, T. L. (2005). Minimum sample size recommendations for conducting factor analyses. *International Journal of Testing,**5*(2), 159–168. 10.1207/s15327574ijt0502_4

[CR59] Niedeggen, M., Wichmann, P., & Stoerig, P. (2001). Change blindness and time to consciousness. *European Journal of Neuroscience*, *14*(10), 1719–1726. 10.1046/j.0953-816X.2001.01785.x 10.1046/j.0953-816x.2001.01785.x 11860466

[CR60] Ozili, P. K. (2023). The acceptable R-square in empirical modelling for social science research. *Social research methodology and publishing results: A guide to non-native English speakers* (pp. 134–143). IGI global. 10.4018/978-1-6684-6859-3.ch009

[CR61] Pailian, H., Simons, D. J., Wetherhold, J., & Halberda, J. (2020). Using the flicker task to estimate visual working memory storage capacity. *Attention, Perception, and Psychophysics,**82*(3), 1271–1289. 10.3758/s13414-019-01809-1 10.3758/s13414-019-01809-1 31321648

[CR62] Pringle, H. L., Irwin, D. E., Kramer, A. F., & Atchley, P. (2001). The role of attentional breadth in perceptual change detection. *Psychonomic Bulletin & Review,**8*(1), 89–95. 11340871 10.3758/bf03196143

[CR63] Rensink, R. A., O’Regan, J. K., & Clark, J. J. (1997). To see or not to see: The need for attention to perceive changes in scenes. *Psychological Science*. 10.1111/j.1467-9280.1997.tb00427.x

[CR64] Rensink, R. A., O’Regan, K., & Clark, J. J. (2000). On the failure to detect changes in scenes across brief interruptions. *Visual Cognition,**7*(1–3), 127–145. 10.1080/135062800394720

[CR65] Rizzo, M., Sparks, J., McEvoy, S., Viamonte, S., Kellison, I., & Vecera, S. P. (2009). Change blindness, aging, and cognition. *Journal of Clinical and Experimental Neuropsychology,**31*(2), 245–256. 10.1080/13803390802279668 19051127 10.1080/13803390802279668 PMC3146260

[CR66] Robison, M. K., & Unsworth, N. (2017). Individual differences in working memory capacity predict learned control over attentional capture. *Journal of Experimental Psychology: Human Perception and Performance,**43*(11), 1912–1924. 10.1037/xhp0000419 28406685 10.1037/xhp0000419

[CR67] Rouder, J. N., & Haaf, J. M. (2019). A psychometrics of individual differences in experimental tasks. *Psychonomic Bulletin and Review,**26*(2), 452–467. 10.3758/s13423-018-1558-y 30911907 10.3758/s13423-018-1558-y

[CR68] Rouder, J. N., Morey, R. D., Morey, C. C., & Cowan, N. (2011). How to measure working memory capacity in the change detection paradigm. *Psychonomic Bulletin and Review,**18*(2), 324–330. 10.3758/s13423-011-0055-3 21331668 10.3758/s13423-011-0055-3 PMC3070885

[CR69] Sareen, P., Ehinger, K. A., & Wolfe, J. M. (2016). CB database: A change blindness database for objects in natural indoor scenes. *Behavior Research Methods,**48*(4), 1343–1348. 10.3758/s13428-015-0640-x 26660198 10.3758/s13428-015-0640-x PMC4902788

[CR70] Schankin, A., Bergmann, K., Schubert, A. L., & Hagemann, D. (2017). The allocation of attention in change detection and change blindness. *Journal of Psychophysiology,**31*(3), 94–106. 10.1027/0269-8803/a000172

[CR71] Scott, W. A. (1962). Cognitive complexity and cognitive flexibility. *Sociometry,**25*(4), 405. 10.2307/2785779

[CR72] Simmons, J. P., Nelson, L. D., & Simonsohn, U. (2012). A 21 word solution. *Available at SSRN 2160588*.

[CR73] Simons, D. J., & Ambinder, M. S. (2005). Change blindness: Theory and consequences. *Current Directions in Psychological Science,**14*(1), 44–48.

[CR74] Simons, D. J., & Levin, D. T. (1998). Failure to detect changes to people during a real-world interaction. *Psychonomic Bulletin & Review,**5*(4), 644–649.

[CR75] Simons, D. J., & Rensink, R. A. (2005). Change blindness: Past, present, and future. *Trends in Cognitive Sciences,**9*(1), 16–20. 10.1016/j.tics.2004.11.006 15639436 10.1016/j.tics.2004.11.006

[CR76] Snijder, J. P., Tang, R., Bugg, J. M., Conway, A. R. A., & Braver, T. S. (2024). On the psychometric evaluation of cognitive control tasks: An investigation with the Dual Mechanisms of Cognitive Control (DMCC) battery. *Behavior Research Methods,**56*(3), 1604–1639. 10.3758/s13428-023-02111-7 37040066 10.3758/s13428-023-02111-7 PMC10088767

[CR77] Souza, A. S., & Oberauer, K. (2017). The contributions of visual and central attention to visual working memory. *Attention, Perception, and Psychophysics,**79*(7), 1897–1916. 10.3758/s13414-017-1357-y 10.3758/s13414-017-1357-y 28600676

[CR78] Stolwyk, R. J., Lee, S., McKay, A., & Ponsford, J. L. (2013). Exploring what the Austin Maze measures: A comparison across conventional and computer versions. *Brain Impairment,**14*(2), 243–252. 10.1017/BrImp.2013.23

[CR79] The jamovi project (2025). *jamovi*. (Version 2.7) [Computer Software]. Retrieved from https://www.jamovi.org

[CR80] Tseng, P., Hsu, T. Y., Muggleton, N. G., Tzeng, O. J. L., Hung, D. L., & Juan, C. H. (2010). Posterior parietal cortex mediates encoding and maintenance processes in change blindness. *Neuropsychologia,**48*(4), 1063–1070. 10.1016/j.neuropsychologia.2009.12.005 20005882 10.1016/j.neuropsychologia.2009.12.005

[CR81] Tu, D., Li, Y., & Cai, Y. (2022). A new perspective on detecting performance decline: A change-point analysis based on Jensen-Shannon divergence. *Behavior Research Methods,**55*(3), 963–980. 10.3758/s13428-021-01779-z 35524039 10.3758/s13428-021-01779-z

[CR82] Vogel, E. K., & Machizawa, M. G. (2004). Neural activity predicts individual differences in visual working memory capacity. *Nature,**428*(6984), 748–751. 10.1038/nature02447 15085132 10.1038/nature02447

[CR83] Vogel, E. K., Woodman, G. F., & Luck, S. J. (2001). Storage of features, conjunctions, and objects in visual working memory. *Journal of Experimental Psychology: Human Perception and Performance,**27*(1), 92–114. 10.1037/0096-1523.27.1.92 11248943 10.1037//0096-1523.27.1.92

[CR84] Waite, S., Farooq, Z., Grigorian, A., Sistrom, C., Kolla, S., Mancuso, A., Martinez-Conde, S., Alexander, R. G., Kantor, A., & Macknik, S. L. (2020). A review of perceptual expertise in radiology-How it develops, how we can test it, and why humans still matter in the era of artificial intelligence. *Academic Radiology,**27*(1), 26–38. 10.1016/j.acra.2019.08.018 31818384 10.1016/j.acra.2019.08.018

[CR85] Woods, D. L., Wyma, J. M., Herron, T. J., & Yund, E. W. (2016). An improved spatial span test of visuospatial memory. *Memory,**24*(8), 1142–1155. 10.1080/09658211.2015.1076849 26357906 10.1080/09658211.2015.1076849

[CR86] Wright, T. J., Roque, N. A., Boot, W. R., & Stothart, C. (2018). Attention capture, processing speed, and inattentional blindness. *Acta Psychologica,**190*, 72–77. 10.1016/j.actpsy.2018.07.005 30016757 10.1016/j.actpsy.2018.07.005 PMC6309252

[CR87] Youmans, R. J., Figueroa, I. J., & Kramarova, O. (2011). Reactive task-set switching ability, not working memory capacity, predicts change blindness sensitivity. *Proceedings of the Human Factors and Ergonomics Society,**55*(1), 914–918. 10.1177/1071181311551190

[CR88] Zorowitz, S., & Niv, Y. (2023). Improving the reliability of cognitive task measures: A narrative review. *Biological Psychiatry: Cognitive Neuroscience and Neuroimaging,**8*(8), 789–797. 10.1016/j.bpsc.2023.02.004 36842498 10.1016/j.bpsc.2023.02.004 PMC10440239

[CR89] Zwierko, T., Lesiakowski, P., Redondo, B., & Vera, J. (2022). Examining the ability to track multiple moving targets as a function of postural stability: A comparison between team sports players and sedentary individuals. *PeerJ,* Article 10. 10.7717/peerj.13964 10.7717/peerj.13964 PMC9443790 36071825

