# Supplementary material for: Subdomains of executive function correlate with accuracy on a change detection task
Source: Psychon Bull Rev. 2026 Jun 8;33(5):171. doi: 10.3758/s13423-026-02937-0 (PMC13246876; doi:10.3758/s13423-026-02937-0)
Supplement: Supplementary file 1 — Supplementary file1 (DOCX 495 KB) [file 13423_2026_2937_MOESM1_ESM.docx]

# Appendix A

**Exploratory Factor Analysis Summary**

Principal Axis Factoring is the most appropriate method when there are violations of multivariate normality (Fabrigar et al., 1999). Promax was chosen as it is an oblique rotation method and factors were expected to be correlated (Howard, 2016). For initial analyses, parallel analysis was used to determine the number of factors to be retained. This method was chosen as it is one of the most accurate methods in deciding how many factors should be retained (Hayton et al., 2004).

The first analysis, which included all variables shown in Table 1 (excluding the AFFT variables), revealed 5 factors, and accounted for 43.58% of the variance. However, an inspection of the pattern matrix showed that one factor, composed of the response times for both the head and tail of the Subtle Cognitive Impairment Test [SCIT] had high loadings, with response time for the head exceeding 1. This high loading was anticipated due to the high correlation between these two variables (*r* = .78). Additionally, the error rate on the tail of the SCIT was deemed unlikely to contribute unique variance as most participants did not commit errors for this portion of the task. Thus, it was decided to only include error rate for the head, and response time for the tail of the SCIT. The Flanker task did not load > .30 on any factor suggesting the variability in performance on this task could not be explained by either of the underlying constructs of attention control, visuospatial working memory, and cognitive flexibility, thus it was removed. Lastly, although the time taken to complete the Austin Maze had a significant weak correlation with AFFT scores, this variable loaded with the mean reaction time on the Brief Psychomotor Vigilance Task, response time on both conditions of the Visual Attentional Capture and Control Task. As participants were instructed to prioritise accuracy over speed, and the number of errors across 10 trials is the main outcome of interest, this variable was also removed.

Removal of these four variables (response time for the head of the SCIT, error rate for the tail of the SCIT, Flanker Arrow Task, and Austin Maze Time Taken) yielded 4 factors, however, the error rate on the head of the SCIT cross-loaded on two factors. Constraining the factor solution to 3 factors provided the simplest factor solution with each factor having a minimum of three variables and minimal cross loading (Costello & Osborne, 2005). The final factor solution was interpreted based on factor loadings greater than .30 and the theoretical understanding of the constructs each cognitive test aimed to measure. Each of the aforementioned factor solutions is shown in the jamovi file (see Supplementary Material).

Both the Kaiser-Meyer-Olkin measure and Bartlett’s test of sphericity verified the data suitability for factor analysis, KMO = .70 (Kaiser, 1974), χ²(78) = 754.30, *p* < .001. Three factors were extracted accounting for 40% of the variance. Factor 1, which accounted for 19% of the variance, was labelled "cognitive flexibility”, consisting of the Wisconsin Card Sorting Test variables (perseverative errors, sets sorted, and correct sorts). Factor 2, which accounted for an additional 12% of the variance, was labelled “visuospatial working memory” due to its high loadings with the Computerised Spatial Span Test tasks, Object 2back Task, Multiple Object Tracking, Austin Maze total errors, and Visual Attentional Capture and Control Task static RT. Lastly, factor 3, which accounted for an additional 10% of the variance, consisted of the Subtle Cognitive Impairment Test error rate for the head and response time for the tail, Visual Attentional Capture and Control Task dynamic RT, and Brief Psychomotor Vigilance Task mean RT. Although it was anticipated that factor 3 would be “attention control”, due to the absence of the Flanker Deadline and Multiple Object Tracking tasks it was instead labelled “attention and processing speed”.

# Appendix B

**Data Analysis Including Response Time on the Alternate Forms Flicker Task**

Table B1

*Descriptive Statistics for all Outcome Variables*

| Variable | *N* | Mean (*SD*) | Range | Skewness | Kurtosis | Reliability |
| --- | --- | --- | --- | --- | --- | --- |
| AFFT Score | 260 | 8.26 (2.96) | 1.00-17.00 | 0.15 | 0.04 | Set A = .40  Set B = .60 |
| AFFT Response Time (ms) | 260 | 21842 (2898) | 13905-29026 | -0.32 | -0.08 | N/A |
| VACCT Dynamic RT (ms) | 210 | 870 (172) | 486-1418 | 0.79 | 0.74 | α = .96 |
| VACCT Static RT (ms) | 206 | 3621 (948) | 1947-6895 | 1.03 | 0.85 | α = .86 |
| PVT-B Mean RT (ms) | 258 | 290 (38) | 201-425 | 0.51 | 0.62 | α = .96 |
| AM Errors | 256 | 39.64 (21.57) | 7.00-112.00 | 0.85 | 0.26 | *r* = .95 |
| AM Total Time Taken | 259 | 257 (75) | 120-494 | 0.86 | 0.53 | *r* = .93 |
| SCIT-RT_H_ (ms) | 259 | 642 (100) | 383-925 | 0.08 | -0.13 | α = .93 |
| SCIT-E_H_ (%) | 258 | 28.53 (14.40) | 0.00-78.00 | 0.49 | 0.49 | α = .76 |
| SCIT-RT_T_ (ms) | 258 | 518 (76) | 373-742 | 0.48 | -0.26 | α = .95 |
| SCIT-E_T_ (%) | 250 | 2.68 (3.79) | 0.00-19.00 | 1.84 | 3.79 | α = .86 |
| Flanker Deadline | 220 | 532 (79) | 380-920 | 1.36 | 3.26 | N/A |
| MOT Average Speed | 256 | 3.75 (1.57) | 0.40-9.00 | 0.49 | 0.00 | N/A |
| C-SST Forward Span | 260 | 6.86 (0.95) | 5.00-9.00 | 0.17 | -0.26 | N/A |
| C-SST Backward Span | 259 | 6.44 (1.10) | 4.00-9.00 | 0.15 | -0.38 | N/A |
| O2B Total | 259 | 74.62 (15.60) | 22.41-98.28 | -1.11 | 1.02 | α = .89 |
| WCST Perseverative Errors | 254 | 9.80 (5.91) | 1.00-28.00 | 0.9 | 0.01 | ICC = .72 |
| WCST Sets Sorted | 258 | 4.33 (1.11) | 1.00-6.00 | -0.34 | -0.14 | ICC = .84 |
| WCST Correct | 257 | 48.50 (6.34) | 28.00-58.00 | -1.07 | 0.76 | *r* = .90 |

* *Note.* AFFT = Alternate Forms Flicker Task. VACCT = Visual Attentional Capture and Control Task. PVT-B = Brief Psychomotor Vigilance Task Mean Reaction Time. AM = Austin Maze. SCIT = Subtle Cognitive Impairment Test. RT_H_ = Response Time Head. E_H_ = Error Rate Head. RT_T_ = Response Time Tail. E_T_ = Error Rate Tail. Flanker Deadline = Final Duration to Respond. MOT = Multiple Objective Tracking. C-SST = Computerised Spatial Span Test. O2B Total = Object 2back % of Trials Correct. WCST = Wisconsin Card Sorting Test. The VACCT was a task introduced later into the original research project; the smaller sample size is due to all participants not completing this task and not due to outlier removal or manual exclusion. AFFT and AM reliability based on split-half coefficients with Spearman Brown correction. PVT-B Mean RT Cronbach’s Alpha based on the first valid 60 trials. Internal consistency was not calculated for adaptive and deadline variables due to the nature of these tasks (Draheim et al., 2022). WCST Perseverative Errors and Sets Sorted based on Intraclass Correlation Coefficients by Chiu and Lee (2021).

Table B6

*Intercorrelations Between Outcome Variables*

|  | 1 | 2 | 3 | 4 | 5 | 6 | 7 | 8 | 9 | 10 | 11 | 12 | 13 | 14 | 15 | 16 | 17 | 18 |
| --- | --- | --- | --- | --- | --- | --- | --- | --- | --- | --- | --- | --- | --- | --- | --- | --- | --- | --- |
| 1. AFFT Score | - |  |  |  |  |  |  |  |  |  |  |  |  |  |  |  |  |  |
| 2. AFFT RT | -.70** | - |  |  |  |  |  |  |  |  |  |  |  |  |  |  |  |  |
| 3. VACCT Dynamic RT | -.06 | .09 | - |  |  |  |  |  |  |  |  |  |  |  |  |  |  |  |
| 4. VACCT Static RT | -.33** | .25** | .25** | - |  |  |  |  |  |  |  |  |  |  |  |  |  |  |
| 5. PVT Mean RT | -.10 | .07 | .40** | .19** | - |  |  |  |  |  |  |  |  |  |  |  |  |  |
| 6. AM Errors | -.26** | .13* | .05 | .22** | -.02 | - |  |  |  |  |  |  |  |  |  |  |  |  |
| 7. AM Total Time Taken | -.15* | .15* | .38** | .28** | .26** | .30** | - |  |  |  |  |  |  |  |  |  |  |  |
| 8. SCIT-RT_H_ | -.10 | .23** | .19** | .21** | .18** | .08 | .26** | - |  |  |  |  |  |  |  |  |  |  |
| 9. SCIT-E_H_ | -.18** | .15* | .21** | .17* | .29** | .19** | .31** | .65** | - |  |  |  |  |  |  |  |  |  |
| 10. SCIT-RT_T_ | -.15* | .23** | .27** | .15* | .18** | .13* | .26** | .78** | .48** | - |  |  |  |  |  |  |  |  |
| 11. SCIT-E_T_ | -.14* | .10 | .16* | .22** | .10 | .30** | .22** | .23** | .40** | .41** | - |  |  |  |  |  |  |  |
| 12. Flanker Deadline | -.06 | .00 | .17* | .13 | .23** | .04 | .24** | .08 | .14* | .15* | .25** | - |  |  |  |  |  |  |
| 13. MOT Average Speed | .15* | -.10 | .01 | -.19** | .04 | -.28** | -.14* | -.07 | -.14* | -.15* | -.20** | -.15* | - |  |  |  |  |  |
| 14. C-SST Forward Span | .11 | -.03 | -.05 | -.26** | .00 | -.23** | -.23** | -.14* | -.11 | -.17** | -.19** | -.11 | .17** | - |  |  |  |  |
| 15. C-SST Backward Span | .12 | -.11 | .01 | -.30** | -.06 | -.30** | -.26** | -.11 | -.08 | -.13* | -.22** | -.23** | .25** | .47** | - |  |  |  |
| 16. O2B Total | .17** | -.06 | -.05 | -.23** | -.09 | -.33** | -.18** | -.11 | -.27** | -.15* | -.26** | -.22** | .35** | .22** | .32** | - |  |  |
| 17. WCST Perseverative Errors | -.11 | .07 | .06 | .16* | .03 | .32** | .20** | .11 | .12 | .14* | .17** | .11 | -.15* | -.13* | -.16** | -.29** | - |  |
| 18. WCST Sets Sorted | .04 | .04 | -.02 | -.15* | -.03 | -.26** | -.15* | -.05 | -.07 | -.09 | -.19** | -.16* | .13* | .09 | .06 | .21** | -.71** | - |
| 19. WCST Correct | .08 | -.01 | -.08 | -.19** | -.07 | -.31** | -.20** | -.13* | -.15* | -.15* | -.23** | -.14* | .12 | .13* | .15* | .23** | -.87** | .78** |

*** p* < .01. * *p* <.05. * Note. AFFT = Alternate Forms Flicker Task. VACCT = Visual Attentional Capture and Control Task. PVT-B = Brief Psychomotor Vigilance Task Mean Reaction Time. AM = Austin Maze. SCIT = Subtle Cognitive Impairment Test. RT_H_ = Response Time Head. E_H_ = Error Rate Head. RT_T_ = Response Time Tail. E_T_ = Error Rate Tail. Flanker Deadline = Final Duration to Respond. MOT = Multiple Objective Tracking. C-SST = Computerised Spatial Span Test. O2B Total = Object 2back % of Trials Correct. WCST = Wisconsin Card Sorting Test.

Table B3

*Intercorrelations Between Change Detection Performance and Executive Function Performance*

| Variable | 1. | 2. | 3. | 4. |
| --- | --- | --- | --- | --- |
| 1. AFFT Score | - |  |  |  |
| 2. AFFT Response Time | -.70** | - |  |  |
| 3. Cognitive Flexibility | .10 | -.04 | - |  |
| 4. Visuospatial Working Memory | .30** | -.22* | .38** | - |
| 5. Attention and Processing Speed | .22* | -.25* | .20** | .34** |

*Note*. ** *p* < .001. * *p* < .01. AFFT = Alternate Forms Flicker Task.

Table B4

*Multiple Linear Regression Coefficients Predicting Alternate Forms-Change Detection Performance from Cognitive Flexibility, Visuospatial Working Memory, and Attention and Processing Speed*

| Variable | B | 95% CI | β | *t* | *p* |
| --- | --- | --- | --- | --- | --- |
| AFFT Scores |  |  |  |  |  |
| Constant | 8.29 | [7.89, 8.69] |  | 41.03 | < .001 |
| Cognitive Flexibility | -0.09 | [-0.54, 0.35] | -0.03 | -0.40 | .67 |
| Visuospatial Working Memory | 0.92 | [0.38, 1.45] | 0.27 | 3.38 | < .001 |
| Attention and Processing Speed | 0.47 | [-0.05, 0.99] | 0.13 | 1.78 | .08 |
| AFFT RT |  |  |  |  |  |
| Constant | 21852 | [21467, 22236] |  | 112.17 | < .001 |
| Cognitive Flexibility | 186 | [-244, 615] | 0.06 | 0.85 | .40 |
| Visuospatial Working Memory | -570 | [-1085, -54] | -0.17 | -2.18 | .03 |
| Attention and Processing Speed | -685 | [-1189, -181] | -0.2 | -2.68 | .008 |

* *Note.* AFFT = Alternate Forms Flicker Task. RT = Response Time (milliseconds).

# References

Chiu, E. C., & Lee, S. C. (2021). Test–retest reliability of the Wisconsin Card Sorting Test in people with schizophrenia. *Disability and Rehabilitation*, *43*(7), 996–1000. https://doi.org/10.1080/09638288.2019.1647295

Costello, A. B., & Osborne, J. (2005). Best practices in exploratory factor analysis: Four recommendations for getting the most from your analysis. *Practical Assessment, Research, and Evaluation*, *10*(1), 7. https://doi.org/10.7275/jyj1-4868

Draheim, C., Pak, R., Draheim, A. A., & Engle, R. W. (2022). The role of attention control in complex real-world tasks. *Psychonomic Bulletin & Review*, *29*(4), 1143–1197. https://doi.org/10.3758/s13423-021-02052-2

Fabrigar, L. R., Wegener, D. T., MacCallum, R. C., & Strahan, E. J. (1999). Evaluating the use of exploratory factor analysis in psychological research. *Psychological Methods*, *4*(3), 272–299. https://doi.org/10.1037/1082-989X.4.3.272

Hayton, J. C., Allen, D. G., & Scarpello, V. (2004). Factor retention decisions in exploratory factor analysis: A tutorial on parallel analysis. *Organizational Research Methods*, *7*(2), 191–205. https://doi.org/10.1177/1094428104263675

Howard, M. C. (2016). A review of exploratory factor analysis decisions and overview of current practices: What we are doing and how can we improve? *International Journal of Human-Computer Interaction*, *32*(1), 51–62. https://doi.org/10.1080/10447318.2015.1087664

# 
